# Phase imaging with an untrained neural network

**DOI:** 10.1038/s41377-020-0302-3

**Published:** 2020-05-06

**Authors:** Fei Wang, Yaoming Bian, Haichao Wang, Meng Lyu, Giancarlo Pedrini, Wolfgang Osten, George Barbastathis, Guohai Situ

**Affiliations:** 1 Shanghai Institute of Optics and Fine Mechanics, Chinese Academy of Sciences, 201800 Shanghai, China; 2 Center of Materials Science and Optoelectronics Engineering, University of Chinese Academy of Sciences, 100049 Beijing, China; 3 Institut für Technische Optik, Universität Stuttgart, Pfaffenwaldring 9, 70569 Stuttgart, Germany; 4 Department of Mechanical Engineering, Massachusetts Institute of Technology, Cambridge, MA 02139-4301 USA; 5 Hangzhou Institute for Advanced Study, University of Chinese Academy of Sciences, 310024 Hangzhou, China

**Keywords:** Imaging and sensing, Optical metrology

## Abstract

Most of the neural networks proposed so far for computational imaging (CI) in optics employ a supervised training strategy, and thus need a large training set to optimize their weights and biases. Setting aside the requirements of environmental and system stability during many hours of data acquisition, in many practical applications, it is unlikely to be possible to obtain sufficient numbers of ground-truth images for training. Here, we propose to overcome this limitation by incorporating into a conventional deep neural network a complete physical model that represents the process of image formation. The most significant advantage of the resulting physics-enhanced deep neural network (PhysenNet) is that it can be used without training beforehand, thus eliminating the need for tens of thousands of labeled data. We take single-beam phase imaging as an example for demonstration. We experimentally show that one needs only to feed PhysenNet a single diffraction pattern of a phase object, and it can automatically optimize the network and eventually produce the object phase through the interplay between the neural network and the physical model. This opens up a new paradigm of neural network design, in which the concept of incorporating a physical model into a neural network can be generalized to solve many other CI problems.

Recently, deep learning (DL) has shown great potential for solving inverse problems in computational imaging (CI)^1^. Pioneering studies have demonstrated the applicability of DL in optical tomography^2^, computational ghost imaging^3,4^, digital holography^5–7^, imaging through scattering media^8–10^, fluorescence lifetime imaging^11^ imaging under low-light conditions^12^, phase imaging^13–15^, unwrapping^16^, and fringe analysis^17^. Generally, an artificial neural network used in CI requires a large set of labeled data to optimize its weight and bias parameters (training) so that it can represent a universal function that maps the data in the object space into the image space^1^. Depending on the network architecture and the amount of data used for training, the network training process can take several hours or even several days, although the reconstruction process is very quick in most cases. Thus, the acquisition of a sufficiently large set of training data is crucial for the training of a good neural network. However, in many applications, one is usually required to image something that has never been seen before. It is thus impossible to acquire sufficient ground-truth images for network training, resulting in limited generalization ability^9,18^.

We demonstrate in this letter that it is possible to experimentally recover an image with an untrained neural network that is built by combining a conventional artificial network such as U-Net^19^ with a real-world physical model that represents the image formation physics; we call the resulting model PhysenNet. Thus, one does not need thousands of labeled data to train PhysenNet before it can be used. Instead, one needs only to feed a single image to be processed into a PhysenNet model with a suitable handcrafted structure, and the network weight and bias factors will be optimized through the interplay between the neural network and the physical model, eventually resulting in a feasible solution that satisfies the imposed physical constraints. The idea of enforcing implicit priors by means of the handcrafted network structure in PhysenNet is inspired by the concept of the deep image prior (DIP)^20^. We note that the DIP alone has been used in some CI applications^20–23^, but all these studies have been largely limited to simulations. The incorporation of the DIP with a task-specific physical model for optical imaging and its demonstration for coherent imaging experiments are the main contributions of this work. Here, we take phase imaging as a typical example to explain the principle more explicitly.

Phase problems are encountered in many applications, ranging from astronomy to industrial inspection. However, phase imaging is a highly ill-posed problem^24^ when relying on intensity-only measurements^25,26^, and sometimes requires a separate reference beam to encode the phase into fringe patterns^27^. The PhysenNet approach proposed here requires only one intensity *I*(*x, y; z* = *d*), which is a diffraction pattern of a phase-only object *ϕ*(*x, y; z* = 0) located at *z* = 0 over a distance *z* = *d*, acquired using a single-beam set-up, i.e., without a separate reference. The basic concept is schematically outlined in Fig. 1a. The diffraction pattern *I*(*x, y; d*) is the only input to PhysenNet, which has a handcrafted structure that is designed to generate an estimate of the phase object, $\tilde \phi (x,y;0)$. In a conventional neural network, the ground-truth phase object *ϕ*(*x, y*; 0) in the training set must be known, and one can calculate the error between *ϕ*(*x, y*; 0) and $\tilde \phi (x,y;0)$ to optimize the weights and biases^1,13–15^. By contrast, PhysenNet does not need the ground-truth phase *ϕ*(*x, y*; 0). Instead, it uses a physical model *H* to calculate a diffraction pattern $\tilde I(x,y;d)$ from $\tilde \phi (x,y;0)$ according to, for example, the Huygens–Fresnel principle^28^ and then uses the error between $\tilde I(x,y;d)$ and the measured *I*(*x, y*; *d*) to optimize the weights and biases via gradient descent. This will force the calculated diffraction pattern $\tilde I$ to converge to the measured pattern *I* as the iterative process proceeds, as schematically shown in Fig. 1b. Throughout this iterative process, the search for the phase converges to a feasible solution, as shown by the simulation results presented in Fig. 1c.

**Fig. 1:**
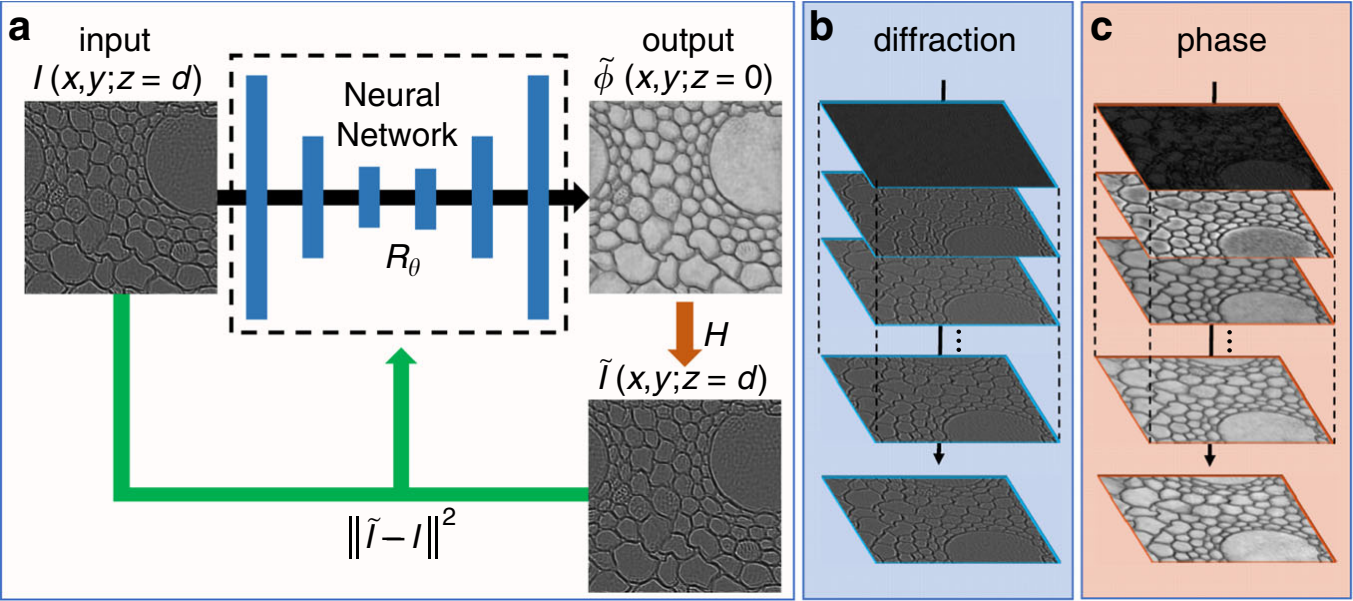
Schematic illustration of the pipeline of PhysenNet. **a** A measured diffraction pattern *I* of a phase object *ϕ* is the input to the neural network. The output of the neural network is taken as the estimated phase $\tilde \phi$, which is then numerically propagated to simulate the diffraction and measurement processes *H* to generate $\tilde I$. We measure the mean square error (MSE) between *I* and $\tilde I$ as the loss value to adjust the neural network parameters *θ*. **b** $\tilde I$ during the optimization process. **c** $\tilde \phi$ during the optimization process

Now, let us take a closer look at the technical details of PhysenNet. For a phase object, *ϕ*(*x, y*; 0), illuminated by a coherent plane wave, the complex amplitude immediately behind it can be written as

1 $$U_0\left( {x,y;0} \right) = \exp \left[ {i\phi \left( {x,y;0} \right)} \right]$$

The diffraction of *U*_0_ over a propagation distance *z* = *d* is given by^28^

2 $$U_d\left( {x,y;d} \right) = {\int} {{\int} {\hat U_0(f_x,f_y){\mathrm{Gexp}}} } \left[ {i2\pi (f_xx + f_yy)} \right]{\mathrm{d}}f_x{\mathrm{d}}f_y$$

where $G = {\mathrm{exp}}\left[ {ikd\sqrt {1 - \lambda ^2f_x^2 - \lambda ^2f_y^2} } \right]$ is the transfer function, $\hat U_0$ is the Fourier transform of *U*_0_, and *f*_*x*_ and *f*_*y*_ are the spatial frequencies in the *x* and *y* directions, respectively. The diffraction pattern recorded by an image sensor can be expressed as

3 $$I\left( {x,y;d} \right) = \left| {U_d(x,y;d)} \right|^2 = H(\phi )$$

where *H*(·) represents the mapping function that relates the phase object *ϕ* to the measured diffraction pattern *I*. The objective of the phase imaging problem is then to formulate an inverse mapping *H*^−1^(·) such that

4 $$\phi \left( {x,y;0} \right) = H^{ - 1}(I\left( {x,y;d} \right))$$

One typical method is to solve the minimization problem $\tilde \phi \left( {x,y} \right) = \mathop{\arg\min}_\phi \|H(\phi ) - I\|^2 + \rho (\phi )$, where *ρ*(*ϕ*) is a handcrafted or dictionary prior^29,30^ that captures the generic regularity of the object, for $\tilde \phi$.

A typical DL-based method is to attempt to learn a mapping function *R* from a large number of labeled data (*ϕ*_*k*_, *I*_*k*_), *k* *=* 1, …, *K*, that form the training set $S_T = \{ \left( {\phi _k,I_k} \right);k = 1, \ldots ,K\}$ by solving

5 $${R}_{\theta ^ \ast } = \mathop{\arg\min}_{\theta \in \Theta }\|R_\theta \left( {I_k} \right) - \phi _k\|^2,\qquad\forall \left( {\phi _k,I_k} \right) \in {S_T}$$

where *R*_*θ*_ is the mapping function of the neural network defined by a set of weights and biases *θ* ∈ Θ. The training process results in a feasible mapping function $R_{\theta ^ \ast }$ that can map a diffraction pattern *I* that is not in *S*_*T*_ back to the corresponding phase $\tilde \phi$, i.e., $\tilde \phi = R_{\theta ^ \ast }(I)$. The size *K* of the training set *S*_*T*_ can be a few thousand or even tens of thousands in a typical CI application^1–17^. Experimentally collecting such a large set of diffraction patterns *I*_*k*_ and their corresponding ground-truth phases *ϕ*_*k*_ is time consuming and usually requires mechanical and environmental stability during the many hours of data acquisition. Although a training set can be created through numerical modeling of the image formation physics^4^, the mapping function learned in such a case works well only for test images that are similar to those in the training set, resulting in good generalization only within the set of objects with the same priors used during training.

Instead, in the PhysenNet model proposed here, the retrieval of the phase is formulated as

6 $$R_{\theta ^ \ast } = \mathop{\arg\min}_{\theta \in \Theta }\|H(R_\theta \left( I \right)) - I\|^2$$

where *H*(·) is defined through the physical model of diffraction described by Eqs. (1)–(3). The ground-truth phase *ϕ* explicitly does not appear in objective function (6), meaning that PhysenNet does not require the ground-truth phase for training. Instead, it is the interplay between *H* and *R*_*θ*_ that causes the prior of *I* to be captured by the handcrafted neural network. When the optimization is complete, the resulting mapping function $R_{\theta ^ \ast }$ can then be used to reconstruct the phase:

7 $$\tilde \phi = R_{\theta ^ \ast }(I)$$

It is worth pointing out that there is no limitation on the network architecture that can be chosen to implement *R*_*θ*_. In our study, we simply adopt U-Net^19^, which has been widely used for CI^1,4,6,7^. Typically, this network structure consists of an encoder path that takes the diffraction pattern as its input, a decoder path that outputs a predicted phase map, and skip paths in the middle. We use four main types of modules to connect the input to the output: convolution blocks (3 × 3 convolution + batch normalization + leaky ReLU), max pooling blocks (2 × 2), up-convolution blocks (3 × 3 de-convolution + batch normalization + leaky ReLU), and skip connection blocks. We use ReLU as the activation function in the output layer. (See Fig. S1 in the Supplementary Information for more details about the architecture.)

The neural network was implemented based on the TensorFlow version 1.9.0 platform using Python 3.6.5. We adopted the Adam optimizer^31^ with a learning rate of 0.01 to optimize the weights and biases, and added uniformly distributed noise between 0 and $\frac{1}{{30}}$ to the fixed input *I* in every optimization step to achieve better convergence^20^. When the training process was complete, we removed the noise and obtained the reconstructed phase in accordance with Eq. (7). In our study, the size of the input image *I* was 256 × 256 pixels. The network usually needed 10,000 epochs to find a very good estimate. This took ~10 min on a computer with an Intel Xeon CPU E5-2696 V3, 64 GB of RAM, and an NVIDIA Quadro P6000 GPU.

We demonstrated the performance of the proposed PhysenNet method through both simulation and experiment. In the simulations, we first compared the proposed method with typical phase retrieval methods, i.e., the Gerchberg–Saxton (GS) algorithm^24,25^ and the transport-of-intensity (TIE) equation^26^. Simulations were conducted using the aforementioned weight parameters. The results are illustrated in Fig. 2. We used the mean square error (MSE) to measure the quality of the reconstructed phase image in comparison to the ground truth shown in Fig. 2a. For a quantitative performance evaluation, we rescaled the reconstructed phases to the same range. The MSE value between the phase reconstructed using PhysenNet (Fig. 2f) and the ground truth is 0.01 rad, whereas the corresponding values associated with the GS algorithm (Fig. 2d) and the TIE equation (Fig. 2e) are 0.03 and 0.06 rad, respectively. In this simulation, PhysenNet used only one diffraction pattern to retrieve the phase, whereas the GS and TIE methods used multiple measurements along the *z* axis as inputs to enhance the quality of the reconstructed phase. In principle, the GS algorithm can retrieve a phase from a single measurement, provided that additional knowledge such as the support of the object is known. However, greater diversity is always preferable^24^.

**Fig. 2:**
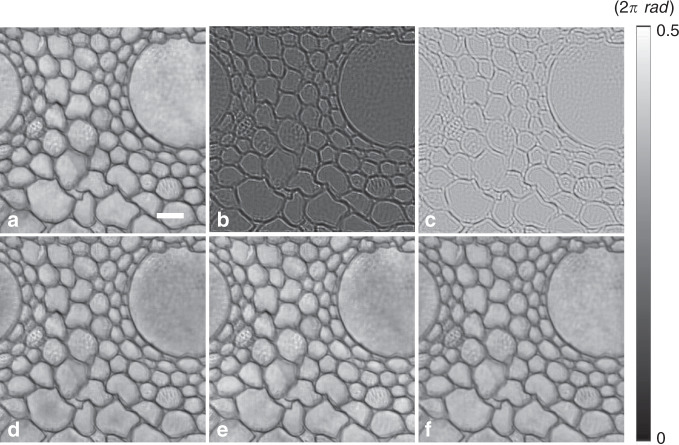
Comparison of different phase imaging methods. **a** The ground-truth phase image: a plant cell slice obtained in our previous work^35^. **b** The diffraction pattern calculated from **a** at *d* = 10 mm. **c**–**f** The phases reconstructed by means of **c** direct reconstruction from **b** via back propagation, **d** the GS algorithm, **e** the TIE equation, and **f** PhysenNet. Scale bar: 256 µm

Next, we numerically analyzed the effect of the diffraction distance *d* on the quality of the reconstructed image. We take three diffraction distances, i.e., *d* = 10 mm, *d* = 95 mm, and *d* = 180 mm, as examples to examine the performance. The results are presented in Fig. 3. One can clearly see from Fig. 3d, e, f, that in all these cases, the phase can be successfully reconstructed from the corresponding diffraction patterns plotted in Fig. 3a–c. This observation is consistent with the reduction in the MSE with an increasing number of epochs that can be seen from the plot in Fig. 3g. Indeed, the MSE values associated with the reconstructed phase maps in Fig. 3d–f with respect to the ground-truth phase image in Fig. 3h are 0.067, 0.061, and 0.076 rad, respectively.

**Fig. 3:**
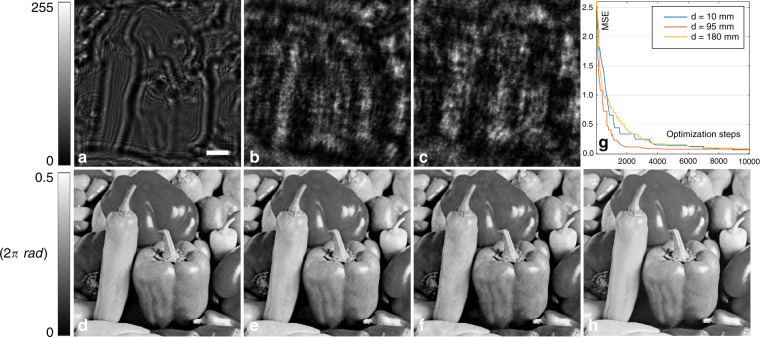
Effect of *d* on the reconstructed phase. The first three images in the top row show the diffraction patterns at *d* values of **a** 10 mm, **b** 95 mm, and **c** 180 mm, and the phase maps reconstructed from these patterns are shown in **d**, **e**, and **f**, respectively. **g** The evolution of the MSE with an increasing number of epochs, and **h** the ground-truth object phase image. Scale bar: 256 µm

We also conducted a direct comparison of PhysenNet and conventional end-to-end approaches for phase imaging. We employed the same neural network structure (without the physical model) to fit the training set (10,000 human face images from Faces-LFW^32^) to obtain a trained model for mapping intensity patterns to phase images (see Table S1 in the Supplementary Information for more details). The results are illustrated in Fig. 4. Again, we used the MSE to measure the quality of the reconstructed phase image in comparison to the ground truth shown in Fig. 4b, which is one of the test images. The MSE value between the phase reconstructed from the diffraction pattern (Fig. 4a) using the pure end-to-end deep learning approach (Fig. 4c) and the ground truth is 0.038 rad, whereas the corresponding value associated with PhysenNet (Fig. 4d) is 0.033 rad. However, we observe that when the phase image is from another set, such as the cat face shown in Fig. 4g, the MSE between the phase reconstructed using the conventional end-to-end approach (Fig. 4h) and the ground truth is 0.127 rad, whereas the corresponding error associated with PhysenNet (Fig. 4i) is 0.025 rad, which is tenfold better. As expected, for the conventional end-to-end deep learning approach, the recovery quality decreases as the similarity between the test object and the training objects decreases. However, the performance of PhysenNet is not similarly affected.

**Fig. 4:**
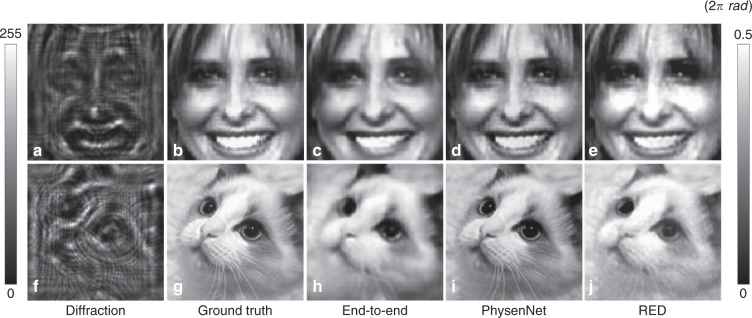
Comparison of PhysenNet, conventional end-to-end DL and RED. **a**, **f** The diffraction patterns at a distance of 10 mm corresponding to the real phase distributions shown in **b** and **g**. **c**, **h** The phase images reconstructed using the conventional end-to-end strategy, **d**, **i** the phase images reconstructed by PhysenNet, and **e**, **j** the phase images reconstructed by RED

We also performed simulations to compare PhysenNet with Regularization by Denoising (RED). We generated the training dataset by adding AWGN (std = 30 dB) to 10,000 images from Faces-LFW^32^. We employed DnCNN^33^ to fit the training dataset to obtain the denoiser for RED. Following^34^, we again used Adam^31^ to minimize the objective as follows:

8 $$\tilde \phi \left( {x,y} \right) = \mathop{\arg\min}_{\phi ,\theta }\|H(R_\theta ^1(I)) - I\|^2 + \frac{\lambda }{2}\phi ^T(\phi - R_{\theta ^ \ast }^2(\phi ))$$

where $R_\theta ^1$ is the deep neural network we used to generate the phase *ϕ* from the diffraction pattern *I*, *λ* is the RED regularization strength, and $R_{\theta ^ \ast }^2$ is the pre-trained denoising model. The results are illustrated in Fig. 4e, j. The MSE values between these results and the ground-truth images are 0.039 and 0.068 rad, respectively.

Now, we will present the experimental demonstration. The experimental apparatus is schematically shown in Fig. 5a. One can see that this is actually a single-beam lens-less imaging geometry. A laser beam emitted from a He–Ne laser at a wavelength of 632.8 nm (NEC Electronics Inc. GLG5002) was first spatially filtered by a pinhole with an aperture of 10 µm and then collimated by a lens with a focal length of *f* = 200 mm. The plane wave was guided to illuminate a phase object, producing intensity images as shown in Fig. 5b. To acquire the diffraction pattern, we placed the camera (SensiCam EM, pixel pitch: 8 µm) at a distance *d* = 22.3 mm from the phase object. The recorded diffraction pattern is shown in Fig. 5c. The proposed PhysenNet takes this diffraction pattern as its only input and generates an output phase map, as shown in Fig. 5d. Off-axis digital holography (DH)^27^ was used to retrieve the object phase image shown in Fig. 5e. As there was only one diffraction pattern available, it was not possible to retrieve the phase by using the TIE equation; however, we did reconstruct the phase from the single diffraction pattern shown in Fig. 5c using the GS algorithm with the phase-only constraint on the object plane, and the result is plotted in Fig. 5f. Note that a separate carrier beam in DH encodes the phase into an intensity pattern, essentially making the phase problem a well-posed one. Here, by taking the DH reconstruction result as the ground truth, we can calculate the MSE between Fig. 5d, e to be 0.084 rad. The cross section highlighted by the dashed line indicates that the phase map reconstructed by the proposed PhysenNet is relatively smooth. In contrast, the MSE between the images reconstructed using the GS and DH methods is 1.926 rad, as clearly evidenced by the noise present in Fig. 5f. Similar observations hold for Fig. 5g–k, which show the results of retrieving the phase for another part of the sample. The MSE value between Fig. 5i and j is 0.093 rad, whereas a value of 2.981 rad is associated with Fig. 5k.

**Fig. 5:**
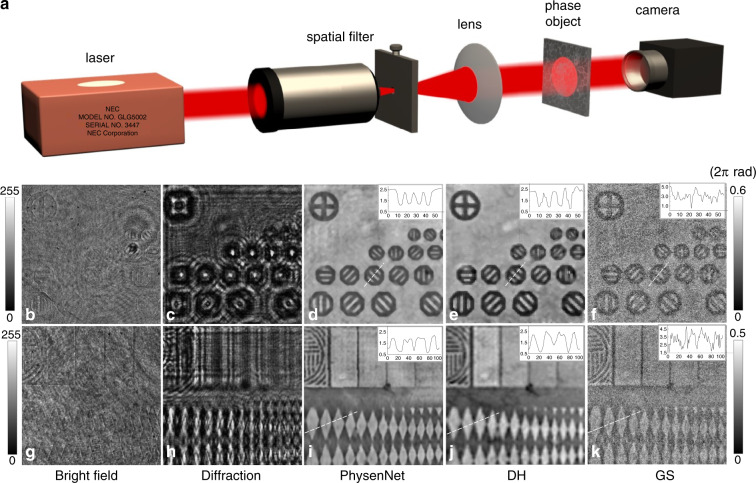
Experimental results. **a** Experimental set-up. **b**, **g** Two different parts of the phase object, **c**, **h** the diffraction patterns, **d**, **i** the phase images reconstructed by PhysenNet, **e**, **j** the phase images reconstructed via off-axis digital holography, and **f**, **k** the phase images reconstructed with the GS algorithm

In all the above investigations, we imposed no assumptions on the profile or support of the phase object, in contrast to almost all other phase retrieval algorithms^24,25^. However, we found that PhysenNet does not work well for phase modulation ranges larger than 2*π*. Resolving this limitation is beyond the scope of the present study.

PhysenNet requires precise modeling of the image formation mechanism [e.g., Eqs. (1)–(3) in our study], and the incorporation of the resulting physical model into a conventional deep neural network (U-Net in our case). It is the interplay between the physical model and the neural network that allows the object phase to be reconstructed with a single intensity measurement. The advantages of PhysenNet in comparison to the pure end-to-end approaches for CI^1^ are straightforward. First, pure end-to-end approaches usually require many labeled data to train a neural network. In physical experiments, such labeled data can be generated by using an SLM, or they can be numerically synthesized using a rigorous image formation model^4^. PhysenNet, on the other hand, does not require any labeled data for training. Instead, all it needs as input is the image to be processed. Second, pure end-to-end approaches learn a mapping function from the statistics of a large set of training data, represented by the weights of the network. When test data are fitted with the same set of weights, test error will inevitably emerge, resulting in artefacts and noise in the reconstructed images, particularly in cases where the test data are far from the training data in terms of their statistics. PhysenNet, inspired by the DIP, does not learn a mapping function from the statistics of the training data but rather is based on the interplay between a handcrafted network structure and a physical image formation model. As a result, the network in PhysenNet is more specifically tuned to perform well in reconstruction from the given input, at the cost of some generalization ability. Although we have demonstrated PhysenNet only for a use case of 2D phase retrieval, it is, in principle, also applicable for 3D objects provided that a multi-projection technique such as tomography is used to collect the data. In these cases, there should be multiple mapping functions *H*_*i*_, where *i* = 1,2, …, *N* denotes the number of projections, that relate the measured intensity *I*_*i*_ to the 3D object function in the *i*th view. These functions *H*_*i*_ should be implemented to represent the associated physics, and objective function (6) should accordingly be generalized to $R_{\theta ^ \ast } = \mathop{\arg\min}_{\theta \in \Theta }\mathop {\sum }\limits_i \|H_i(R_\theta \left( I_i \right)) - I_i\|^2$.

In comparison to conventional DL approaches for CI, the only extra ingredient that PhysenNet needs is a known forward mapping function *H*, as described in Eq. (6). This means that, given an estimate $\tilde U$ of an object function, PhysenNet requires the calculability of the forward transform of $\tilde U$ through an imaging system specified by *H*, which is required to evaluate the cost function. No additional requirements are imposed on either the method of data acquisition or the illumination conditions. As a result, PhysenNet should be applicable for diverse imaging modalities, provided that the forward mapping function is known.

## Supplementary information

Supplementary Information
