# Supplementary material for: Phase imaging with an untrained neural network
Source: Light Sci Appl. 2020 May 6;9:77. doi: 10.1038/s41377-020-0302-3 (PMC7200792; doi:10.1038/s41377-020-0302-3)
Supplement: Supplementary file 1 — Supplementary Information [file 41377_2020_302_MOESM1_ESM.docx]

**Supplementary Information for**

**Phase imaging with an untrained neural network**

Fei Wang^1,2^, Yaoming Bian^1,2^, Haichao Wang^1,2^, Meng Lyu^1,2^, Giancarlo Pedrini^3^, Wolfgang Osten^3^, George Barbastathis^4^, and Guohai Situ^1,2,5^

1. *Shanghai Institute of Optics and Fine Mechanics, Chinese Academy of Sciences, Shanghai, 201800, China*

2. *Center of Materials Science and Optoelectronics Engineering, University of Chinese Academy of Sciences, Beijing 100049, China*

3. *Institut für Technische Optik, Universität Stuttgart, Pfaffenwaldring 9, 70569 Stuttgart, Germany*

4. *Department of Mechanical Engineering, Massachusetts Institute of Technology, Cambridge, Massachusetts 02139-4301, USA*

5. *Hangzhou Institute for Advanced Study, UCAS, Hangzhou 310024, China*

Fig. S1. Architecture of neural network structure.

Table S1. Comparison of conventional pure end-to-end deep learning approach and PhysenNet.

| Methods  Comparison items | Deep learning | PhysenNet |
| --- | --- | --- |
| Explicit Prior | None | None |
| Physical Model | None | Diffraction |
| Dataset | Train: 10,000 diffraction - phase pairs  Test: 1 diffraction pattern | 1 diffraction pattern |
| Number of Iteration | Train: 100,000  Test: 1 | 10,000 |
| Computation Time | Train: ~ 4 hours  Test: ~1 ms | ~10 minutes |
| Performance | Similar: good  Much difference: bad | good |
